# Perinatal factors contributing to chronic kidney disease in a cohort of Japanese children with very low birth weight

**DOI:** 10.1007/s00467-020-04791-1

**Published:** 2020-10-17

**Authors:** Osamu Uemura, Kenji Ishikura, Tetsuji Kaneko, Daishi Hirano, Yuko Hamasaki, Masao Ogura, Naoaki Mikami, Yoshimitsu Gotoh, Takeshi Sahashi, Naoya Fujita, Masaki Yamamoto, Satoshi Hibino, Masaru Nakano, Yasuhiro Wakano, Masataka Honda

**Affiliations:** 1 Department of Clinical Medicine, Japanese Red Cross Toyota College of Nursing, Toyota, Japan; 2 Department of Pediatrics, Ichinomiya Medical Treatment & Habilitation Center, 1679-2 Tomida-nagaresuji, Ichinomiya-city, Aichi 494-0018 Japan; 3 Department of Neonatology and Pediatrics, Nagoya City University Graduate School of Medical Science, Nagoya, Japan; 4 Division of Nephology and Rheumatology, National Center for Child Health and Development, Tokyo, Japan; 5 Department of Pediatrics, Kitasato University School of Medicine, Sagamihara, Japan; 6 Department of Clinical Research, Tokyo Metropolitan Children’s Medical Center, Fuchu, Japan; 7 Department of Pediatrics, The Jikei University School of Medicine, Tokyo, Japan; 8 Department of Nephrology, Toho University Faculty of Medicine, Tokyo, Japan; 9 Department of Pediatric Nephrology, Tokyo Metropolitan Children’s Medical Center, Tokyo, Japan; 10 Department of Pediatric Nephrology, Japanese Red Cross Nagoya Daini Hospital, Nagoya, Japan; 11 Department of Pediatrics, Ichinomiya Municipal Hospital, Ichinomiya, Japan; 12 Department of Pediatric Nephrology, Aichi Children’s Health and Medical Center, Nagoya, Japan; 13 Department of Pediatrics, Seirei Hamamatsu General Hospital, Hamamatsu, Japan; 14 Department of Pediatrics, Toyohashi Municipal Hospital, Toyohashi, Japan

**Keywords:** Very low birth weight, Chronic kidney disease, Prematurity, Intrauterine growth restriction, Neonatal events, Maternal smoking

## Abstract

**Background:**

Developmental programming of chronic kidney disease (CKD) in young adults is linked to preterm birth and intrauterine growth restriction (IUGR). Which confers a higher risk of progression to chronic kidney damage in children with very low birth weight (VLBW; born weighing < 1500 g): prematurity or IUGR?

**Methods:**

This is a national historical cohort study of children with VLBW cared for in perinatal medical centers in Japan. Predictive factors included three latent variables (prematurity, IUGR, stress during neonatal period) and eight observed variables (gestational age, birth weight Z-score, maternal age, duration of treatment with antibiotics and diuretics, maternal smoking, late-onset circulatory collapse, kidney dysfunction) during the perinatal period. The primary endpoint was estimated glomerular filtration rate (eGFR) at age ≥ 3 years. A structural equation model was used to examine the pathologic constitution.

**Results:**

The 446 children with VLBW included 253 boys and 193 girls, of mean age 5.8 ± 2.6 years and mean eGFR 111.7 ml/min/1.73 m^2^ at last encounter. Pathway analyses showed intrauterine malnutrition (*β* = 0.85) contributed more to chronic kidney damage than stress during the neonatal period (*β* = − 0.19) and prematurity (*β* = 0.12), and kidney dysfunction and late-onset circulatory collapse were important observed variables in stress during the neonatal period.

**Conclusions:**

IUGR was more harmful to future kidneys of VLBW neonates. Neonatal kidney dysfunction and late-onset circulatory collapse were important risk factors for subsequent CKD development. This emphasizes the need for obstetricians to monitor for fetal growth restriction and neonatologists to minimize neonatal stress to prevent CKD in later life.

## Introduction

Intrauterine growth restriction (IUGR), low birth weight (LBW), and premature birth have causal relationships to the origins of hypertension, coronary heart disease, and non-insulin-dependent diabetes in men and women [1, 2]. These associations were shown to be independent of the duration of gestation and must therefore be the result of slow fetal growth [3–8]. Subsequently, the hypothesis of the developmental origins of health and disease (DOHaD) has combined the results of experimental, clinical, epidemiological, and public health studies to determine whether various events during early life, including those occurring in utero, are associated with later risks of morbidity, especially of non-communicable chronic diseases [9–11].

Extremely preterm birth itself may be a risk factor for future chronic kidney disease (CKD) [12]. Moreover, childhood-onset CKD [13] and high systolic blood pressure [14] in adolescents are frequent occurrences with a history of LBW or premature birth. Although kidneys continue to form postnatally in preterm neonates, glomerulogenesis ceases after 40 days [15]. In addition, very low birth weight (VLBW; defined as infants born weighing < 1500 g) and prematurity were shown to promote the development of secondary focal segmental glomerulosclerosis (FSGS) [16, 17].

Taken together, these findings indicate an association between VLBW and future CKD. However, preterm delivery might have been shown to not be a direct cause of future CKD [18, 19]. Rather, the intrauterine environment and/or postnatal stress may directly influence future CKD [20–23]. To assess whether neonatal prematurity or IUGR is more harmful, and to determine how various neonatal stresses adversely affect future kidneys, the present nationwide retrospective survey of VLBW infants in Japan assessed factors influencing CKD in childhood.

## Methods

### Study design and population

This was a nationally representative historical cohort study, not structured to determine the prevalence of CKD, and conducted by the Committee of Measures for Pediatric CKD of the Japanese Society for Pediatric Nephrology (JSPN). The first survey was sent in December 2017 to 399 general and local perinatal medical centers in Japan, inviting them to report pediatric patients over 3 years with VLBW who were managed as of April 1, 2017. The second survey was sent in June 2018 to 113 institutions that had participated in the first survey. The deadlines for returning the first and second surveys were March and September 2018, respectively, although the deadline for the second survey was postponed to February 2019.

The first questionnaire was designed to record the approximate number of patients per year with VLBW in each institution, and the willingness to complete the second questionnaire. Factors recorded for each patient during the perinatal or neonatal period included date of birth, sex, gestational age, birth weight and height, maternal age, maternal smoking, gravidity, maternal steroid administration, 5-min Apgar score, pH, respiratory distress syndrome, artificial breathing management period, patent ductus arteriosus, doses of indomethacin, late-onset circulatory collapse treated with corticosteroids, kidney dysfunction (serum creatinine ≥ 1.5 mg/dL) in neonatal period, duration of treatment with antibiotics and diuretics, and duration of enteral feeding. Factors recorded on the last day of consultation in children aged > 3 years included height, weight, blood pressure, proteinuria, serum creatinine (SCr) concentration, serum cystatin C concentration, congenital anomalies of the kidney and urinary tract, confirmed kidney disease, malformation syndrome, congenital heart disease and its severity, digestion or absorption disorder, chromosomal abnormalities, neuromuscular disease, and thyroid disease. For the purposes of this survey, height and weight were recorded within 3 months, and blood pressure and proteinuria within 1 year, after or before measurement of SCr. Age was calculated from the date of birth and the date of last encounter. The respondents were asked to search their medical records for patients with VLBW and with measurements of SCr and height over 3 years. Children with VLBW were included if they were aged over 3 years on April 1, 2017, and if height was measured within 3 months after SCr. If a patient was assessed more than once, the results obtained at the oldest age, but under 19 years, were included.

The study was conducted in accordance with the ethical principles in the Declaration of Helsinki, and with the ethical guidelines for medical and health research involving human subjects stipulated by the Ministry of Education, Culture, Sports, Science and Technology and the Ministry of Health, Labour and Welfare in Japan. The study was approved by the ethics committees of the Japanese Red Cross Toyota College of Nursing (approval number 2911), which waived the requirement for informed consent due to the retrospective nature of this study.

Data were stored in DATASELECT Inc (Aichi, Japan), a data center independent of our study group. Data on personal information were submitted by each hospital as case questionnaires, transferred to an Excel sheet, and stored in the data center. The list of patients was kept in each hospital carefully, preventing investigators from accessing any personal information. The encrypted data sheet was accessible only to a principal investigator and co-investigators.

### Patient and public involvement statement

There has been no patient and public involvement in this study.

### Statistical analysis and variables

Univariable analyses were initially performed only to determine the factors to be entered into multivariable analyses, such as multiple regression analysis and structural equation modeling (SEM). SEM was chosen because it is a powerful statistical modeling technique for observational data that can be used to statistically confirm a hypothesized model by evaluating the observed covariance structure of the data. The initial model was based on the hypothesis that in VLBW infants, prematurity, IUGR, and various stressors during the neonatal period are related to CKD in childhood and adolescence.

In SEM, the opposite of an observed variable is a latent variable. Latent variables are loosely documented events that cannot be observed directly but only implied indirectly through consequences of observed variables, much representing concepts. Although observed variables are the only type of variable used in regression analyses, SEM can handle other types of variables, including latent, unobserved, and theoretical variables. Observed variables are represented by rectangular nodes in SEM, and latent variables are represented by circles or ellipses. An important difference between these two types of variables is that an observed variable usually has a measurement error associated with it, while a latent variable does not. In this study, prematurity, IUGR, and stress in the neonatal period were chosen as latent variables, and gestational age, Z-score of birth weight, maternal age, antibiotic dosing period, diuretic dosing period, maternal smoking, late-onset circulatory collapse, and kidney dysfunction in the neonatal period were selected as observed variables. The definition of a Z-score in this text is the position of a raw score in terms of its distance from the mean, measured in standard deviation units. The double-headed curved arrows connecting two variables represent covariances. We posited the unanalyzed correlations considering clinical impressions and by exploring the model like any other. This helped to improve the overall model fitting.

The primary outcome was eGFR calculated based on SCr at age > 3 years. eGFR was calculated using Uemura’s formula [24] for Japanese children. Because urine protein to creatinine ratio (u-P/C) was available for only 75 (16.8%) patients, u-P/C could not be a study outcome. During initial univariable analyses, *t* tests for independent variables and linear regression analyses for continuous independent variables were used to compare differences in eGFR. For categorized variables, extremely preterm infants were defined as those born at < 28 weeks’ gestation, and extremely low birth weight (ELBW) infants were defined as those with < 1000 g birth weight. Multivariable analyses included factors such as gestational age, weight Z-score, maternal age, antibiotic dosing period, diuretic dosing period, maternal smoking, late-onset circulatory collapse, and kidney dysfunction in the neonatal period. Logistic regression analysis was performed with the same explanatory variables and CKD (eGFR < 83.5 ml/min/1.73 m^2^) as the objective variables, despite the disadvantage of only 30 events.

SEM has the ability to include latent variables, which are loosely documented events assessed by clustering of measured indicator variables. If the initial model fit was poor, the model was improved by adding or subtracting certain paths. Adequate model fit was defined as a comparative fit index (CFI) > 0.90, a root-mean-square error of approximation (RMSEA) < 0.05, and a chi-square mean/degree of freedom (CMIN/df) < 3.0. All statistical analyses were performed using SPSS Statistics 26 (IBM Corporation) and SPSS Amos 26 (IBM Corporation) software.

Complete data with no missing values is needed for many kinds of calculations such as regression analysis. We created the complete dataset by using listwise deletion which removed from our data any observation which had a missing value among one or more variables. Multiple imputation is a simulation-based statistical technique for handling missing data. After multiple imputation by SPSS, multiple regression analysis, logistic regression analysis, and SEM in the imputed dataset were performed. Little’s MCAR test showed that the null hypothesis (data missing completely at random) was rejected (chi-squared = 39.890, df = 26, *p* = 0.040). Multivariable analyses of the complete dataset showed that the results from the two datasets resembled each other closely.

## Results

### Subject characteristics

**Fig. 1:**
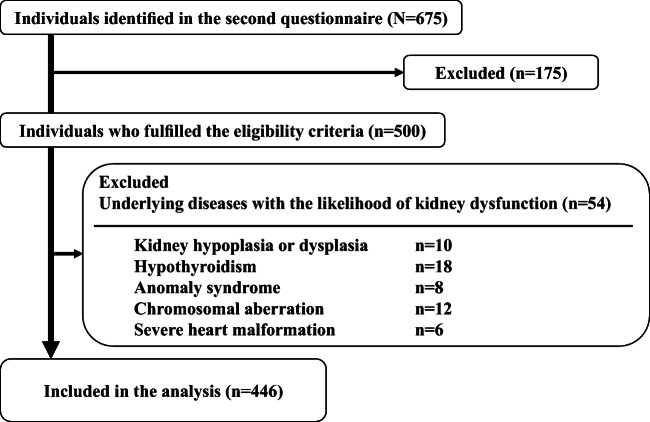
Flowchart illustrating the inclusion/exclusion of individuals in the study. The second questionnaire identified 675 children at 15 institutions. Of these, 446 children, 253 boys and 193 girls, with VLBW fulfilled the eligibility criteria and were included in this study

Of the 399 institutions sent the first questionnaire, 218 (54.6%) responded. The second questionnaire identified 675 children at 15 institutions. Of these, 446 VLBW children, 253 boys and 193 girls, who fulfilled the eligibility criteria were included in this study (Fig. 1). Their demographic and clinical characteristics are summarized in Table 1. The *Z*-scores of birth weight were lower than those of head circumference (HC), and the mean ± SD of HC/birth weight ratio (HC/BW) was 0.028 ± 0.008 (cm/g; *n* = 398). The correlation coefficient between HC/BW and eGFR at last encounter was − 0.113 (*p* = 0.025), showing very weak negative correlation. At last encounter, the mean ± SD age of these children was 5.8 ± 2.6 years and their mean eGFR was 111.7 ml/min/1.73 m^2^. Only one child (0.2%) had stage 3 CKD (eGFR, 55.7 ml/min/1.73 m^2^), whereas 30 (6.7%) had abnormal kidney function, as shown by eGFR < 83.5 ml/min/1.73 m^2^ [25].

**Table 1:** Demographic and clinical characteristics of patients included in the analysis

	Number	Missing values	Average	Standard deviation	Minimum	Maximum
Neonate						
Maternal age (years)	413	33	31.9	5.6	15	51
Gestational age (weeks)	443	3	28.8	3.3	22.0	37.3
Birth weight (g)	446	0	977	311	306	1496
* Z*-score	443	3	− 1.34	1.53	− 5.75	1.85
Birth height (cm)	418	28	34.8	4.3	22.5	47.1
* Z*-score	415	31	− 0.98	1.44	− 5.14	3.39
Head circumference (cm)	398	48	25.3	2.9	13.5	38.8
* Z*-score	396	50	− 0.42	1.26	− 5.08	8.23
5-min Apgar score	432	14	7.1	2.0	1	10
pH at birth	422	24	7.30	0.11	6.6	7.6
Hb at birth	421	25	15.3	2.9	4.2	24.8
Duration of mechanical ventilation (days)	416	30	48.0	60.3	0	616
Duration of oxygen administration (days)	431	15	63.3	85.9	0	848
Establishment of enteral nutrition (day)	400	46	15.5	24.4	0	374
Last encounter						
Age (years)	446	0	5.8	2.6	3	16
eGFR	446	0	111.7	21.6	55.7	212.8

The definition of a *Z*-score is the position of a raw score in terms of its distance from the mean, measured in standard deviation units

### Univariable analyses and variables chosen for multivariable analyses

Table 2 shows the results of univariable analyses of categorized variables, with eGFR as a dependent variable. Linear regression analyses showed that antibiotic dosing period (*R*^2^ = 0.306, *p* = 0.003) and diuretic dosing period (*R*^2^ = 0.779, *p* = 0.000) correlated significantly with eGFR. Based on these results and our clinical impressions, our multivariable analyses included exposure variables, such as gestational age, weight *Z*-score, maternal age, antibiotic dosing period, diuretic dosing period, maternal smoking, late-onset circulatory collapse, and kidney dysfunction in the neonatal period.

**Table 2:** Results of univariable analyses with eGFR as a dependent variable.

	Number	Mean (eGFR)	SD (eGFR)	*p* value
Gestational age				
≥ 28 weeks	254	112.4	21.8	0.392
< 28 weeks	189	110.6	21.4	
Birth weight				
≥ 1000 g	206	113.7	23.1	0.062
< 1000 g	240	109.9	20.2	
*Z*-score of birth weight				
≥ − 2.0	295	113.2	22.9	0.038
< − 2.0	148	108.7	18.4	
Maternal age				
≥ 35	131	110.0	22.6	0.175
< 35	282	113.1	21.0	
Maternal smoking				
Yes	28	103.8	21.1	0.038
No	282	112.5	19.9	
Late circulatory collapse required steroids				
Yes	55	105.5	14.4	0.004
No	378	112.2	22.2	
Neonatal kidney dysfunction (SCr ≥ 1.5 mg/dl)				
Yes	43	103.6	20.1	0.011
No	374	112.5	21.9	

The definition of a *Z*-score is the position of a raw score in terms of its distance from the mean, measured in standard deviation units

### Multiple regression analysis

Multivariable analyses of the imputed dataset (Table 3) showed that the *Z*-score of birth weight (standardized coefficient = 0.157, *p* = 0.007), maternal smoking (standardized coefficient = − 0.115, *p* = 0.014), and neonatal kidney dysfunction (standardized coefficient = − 0.112, *p* = 0.021) were independently associated with eGFR in children aged > 3 years. Multivariable analyses of the complete dataset also showed that the *Z*-scores of birth weight (standardized coefficient = 0.203, *p* = 0.017) and neonatal kidney dysfunction (standardized coefficient = − 0.157, *p* = 0.028) were independently associated with eGFR, whereas maternal smoking (standardized coefficient = − 0.080, *p* = 0.235) was not.

**Table 3:** Results of the multiple regression analysis with eGFR as a dependent variable.

	Unstandardized coefficient		Standardized coefficient	
	*B*	Standard error	*β*	*P* value
(Intercept)	111.533	13.484		0.000
Gestational age	0.537	0.432	0.082	0.214
*Z*-score of birth weight	2.223	0.821	0.157	0.007
Maternal age	− 0.316	0.181	− 0.082	0.082
Maternal smoking	− 8.274	3.351	− 0.115	0.014
Late circulatory collapse required steroids	− 5.755	3.305	− 0.090	0.082
Neonatal kidney dysfunction (SCr ≥ 1.5 mg/dl)	− 8.015	3.457	− 0.112	0.021
Antibiotic dosing periods	− 0.018	0.068	− 0.013	0.787
Diuretic dosing periods	0.017	0.020	0.041	0.404

The definition of a *Z*-score is the position of a raw score in terms of its distance from the mean, measured in standard deviation units

### Logistic regression analysis

Logistic regression analysis of the imputed dataset showed only maternal smoking was independently associated with CKD (eGFR < 83.5 ml/min/1.73 m^2^) with odds ratio of 2.89 (95% confidential interval: 1.13–7.35, *p* = 0.026). However, the other seven factors did not have significant odds ratios.

### Structural equation modeling

**Fig. 2:**
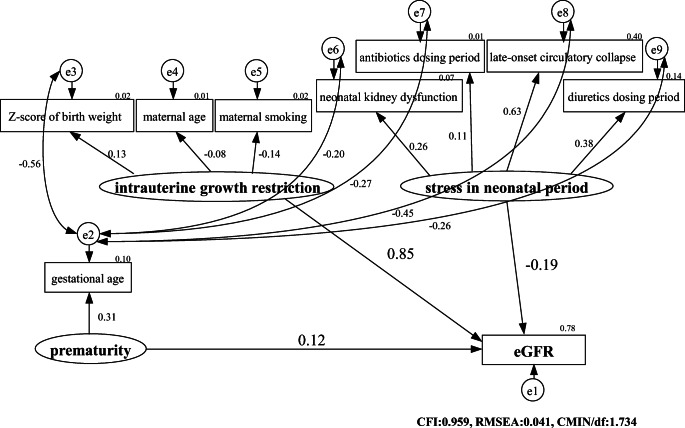
Results of the structural equation model with eGFR as a dependent variable. The model fit in the pathway analyses was adequate (CFI 0.959, RMSEA 0.041, and CMIN/df 1.734). The pathways were determined using exploratory methods, in addition to the standardized regression coefficients. IUGR (*β* = 0.85) contributed more to childhood kidney damage than did stress during the neonatal period (*β* = -0.19) and prematurity (*β* = 0.12). Gestational age may be an indicator of prematurity; the *Z*-score of birth weight, maternal age, and maternal smoking may be indicators of IUGR; and antibiotic dosing periods, diuretic dosing periods, late-onset circulatory collapse, and neonatal kidney dysfunction may be indicators of neonatal stress. The definition of a *Z*-score is the position of a raw score in terms of its distance from the mean, measured in standard deviation units.

The model fit in the pathway analyses was adequate (CFI 0.959, RMSEA 0.041, and CMIN/df 1.734). The pathways determined using the exploratory methods, as well as their standardized regression coefficients, are depicted in Fig. 2. Pathway analyses of the imputed dataset showed that, of the latent variables, IUGR (*β* = 0.85) contributed more to kidney damage than stress during the neonatal period (*β* = − 0.19) and prematurity (*β* = 0.12). Gestational age may be an indicator of prematurity; *Z*-score of birth weight, maternal age, and maternal smoking may be indicators of IUGR; and antibiotic dosing period, diuretic dosing period, late-onset circulatory collapse, and neonatal kidney dysfunction may be indicators of stress during the neonatal period. Analysis of the complete dataset also showed that IUGR (*β* = 0.81) contributed more to kidney damage than stress during the neonatal period (*β* = − 0.15) and prematurity (*β* = 0.11), results similar to those in the imputed dataset.

## Discussion

This historical cohort study of 446 children with VLBW who were surveyed at general and local perinatal medical centers found that IUGR contributed more to reduced kidney function in childhood than did prematurity and stress during the neonatal period, with neonatal stress contributing more to reduced kidney function than prematurity. Factors indicative of stress during the neonatal period included late-onset circulatory collapse and kidney dysfunction. We demonstrated that prematurity was less associated with future kidney function than IUGR in patients with VLBW, which concurs with previous research [18–21, 26]. The reason for adopting SEM as well as multiple regression for statistical analysis was to clarify the disease pathogenesis of subsequent CKD development in patients born with VLBW. The disease structure is shown visually in Fig. 2. The path diagram and standardized coefficients (*β*) shown may represent the disease structure. In logistic regression analysis, only maternal smoking was associated with CKD (eGFR < 83.5 ml/min/1.73 m^2^); however, a larger scale study with the appropriate number of events is recommended in order to be conclusive.

In assessing whether neonatal prematurity or IUGR was more harmful to future kidneys, we found that IUGR contributed more to kidney function deterioration during childhood than did prematurity. We considered from the head circumference analysis that the IUGR in this study was predominantly indicative of fetal malnutrition. Low birth weight was shown to be associated with coronary heart disease in both men and women [2]. Because this association was independent of the duration of gestation, it must be the result of slow fetal growth [3–8]. Although the types of disease are different, the present study found that slow fetal growth had a greater effect than the duration of gestation on the onset of childhood CKD. By the way, premature births or low birth weights were considered strong risk factors for the development of CKD from childhood into adulthood [26, 27]. Our results were based on the consideration of prematurity, IUGR, and neonatal stresses and could indicate that pregnant women should be better educated on proper diet, sufficient nourishment, and the importance of not smoking.

The adverse effects of various neonatal stresses on future kidneys were also unclear. Stress during the neonatal period was found to have a more deleterious effect than prematurity, with factors such as late-onset circulatory collapse and kidney dysfunction being important factors that affect subsequent kidney function. It is probable that acute kidney injury is a harbinger for CKD in pediatric populations and our study supports this concept [28]. Longer duration of antibiotic or diuretic treatment had a detrimental effect on kidney function. Postnatal development is affected by acute kidney injury as well as exposure to nephrotoxins such as gentamicin and non-steroidal anti-inflammatory drugs [15]. Late-onset circulatory collapse may also affect postnatal kidney development.

This study had several limitations. First, only 54.6% of the surveyed institutions responded to the first questionnaire, and only 675 children in 15 institutions were identified in the second questionnaire, which may limit the accuracy of our findings. Because this study was not an epidemiologic but a risk factor study, we believe that the homogeneity of research objects was not essential. Second, although eGFR was calculated based on enzymatically measured SCr concentrations [24], including reference SCr concentrations in Japanese children [29], and height, this formula is relatively inaccurate in children with small or large muscle mass. Although children with hypothyroidism, anomaly syndrome, chromosomal aberrations, and severe heart malformation were excluded, the correspondence observed may be inaccurate. Third, subjects underwent final assessment during childhood or adolescence, despite the true endpoint being the evaluation of CKD in adulthood. Although the latter could not be performed at this time, further follow-up may reveal the association between VLBW and adult CKD. Some children might have CKD progression after puberty. Finally, the retrospective design of this study prevented our obtaining sufficient urinalysis results to evaluate.

To our knowledge, this is the first exploratory survey of children with VLBW to determine factors in utero, birth, and neonatal period associated with CKD in childhood, using the structural equation model. IUGR was more harmful to the future kidneys of VLBW neonates. Neonatal kidney dysfunction and late-onset circulatory collapse had adverse effects on subsequent development of CKD. This emphasizes the need for obstetricians to monitor for fetal growth restriction and neonatologists to minimize neonatal stress to prevent CKD in later life.

## Data Availability

Not applicable.
